# Triglyceride-glucose index as a potential predictor for in-hospital mortality in critically ill patients with intracerebral hemorrhage: a multicenter, case–control study

**DOI:** 10.1186/s12877-024-05002-4

**Published:** 2024-05-01

**Authors:** Yang Yang, Shengru Liang, Jiangdong Liu, Minghao Man, Yue Si, Dengfeng Jia, Jianwei Li, Xiaoxi Tian, Lihong Li

**Affiliations:** 1 Department of Emergency, Tangdu Hospital, Fourth Military Medical University, Xi’an, 710038 China; 2 Department of Endocrinology, Tangdu Hospital, Fourth Military Medical University, Xi’an, 710038 China; 3 Department of Neurosurgery, Tangdu Hospital, Fourth Military Medical University, Xi’an, 710038 China

**Keywords:** Intracerebral hemorrhage, Triglyceride-glucose index, Intensive care unit, All-cause mortality

## Abstract

**Background:**

The correlation between the triglyceride-glucose index (TyG) and the prognosis of ischemic stroke has been well established. This study aims to assess the influence of the TyG index on the clinical outcomes of critically ill individuals suffering from intracerebral hemorrhage (ICH).

**Methods:**

Patients diagnosed with ICH were retrospectively retrieved from the Medical Information Mart for Intensive Care (MIMIC-IV) and the eICU Collaborative Research Database (eICU-CRD). Various statistical methods, including restricted cubic spline (RCS) regression, multivariable logistic regression, subgroup analysis, and sensitivity analysis, were employed to examine the relationship between the TyG index and the primary outcomes of ICH.

**Results:**

A total of 791 patients from MIMIC-IV and 1,113 ones from eICU-CRD were analyzed. In MIMIC-IV, the in-hospital and ICU mortality rates were 14% and 10%, respectively, while in eICU-CRD, they were 16% and 8%. Results of the RCS regression revealed a consistent linear relationship between the TyG index and the risk of in-hospital and ICU mortality across the entire study population of both databases. Logistic regression analysis revealed a significant positive association between the TyG index and the likelihood of in-hospital and ICU death among ICH patients in both databases. Subgroup and sensitivity analysis further revealed an interaction between patients' age and the TyG index in relation to in-hospital and ICU mortality among ICH patients. Notably, for patients over 60 years old, the association between the TyG index and the risk of in-hospital and ICU mortality was more pronounced compared to the overall study population in both MIMIC-IV and eICU-CRD databases, suggesting a synergistic effect between old age (over 60 years) and the TyG index on the in-hospital and ICU mortality of patients with ICH.

**Conclusions:**

This study established a positive correlation between the TyG index and the risk of in-hospital and ICU mortality in patients over 60 years who diagnosed with ICH, suggesting that the TyG index holds promise as an indicator for risk stratification in this patient population.

**Supplementary Information:**

The online version contains supplementary material available at 10.1186/s12877-024-05002-4.

## Introduction

Spontaneous, nontraumatic, intracerebral hemorrhage (ICH) is a catastrophic disease making up approximately 10–20% of all types of stroke [1]. Epidemiological data indicate that 30% of ICH patients requiring intensive care unit (ICU) management and 40% of them die within 30 days [2]. Despite ongoing research and advancements in this medical field, effective therapeutic options for improving the prognosis of patients with ICH are still lacking [3]. Consequently, there is an urgent need to identify remediable factors that may impact the outcomes of ICH, as this information could potentially lead to the development of new therapeutic targets.

Insulin resistance (IR), a pathological condition where tissue does not respond normally to insulin stimulation, plays a crucial role in the development of metabolic disorders [4]. More importantly, studies have revealed that compared with peripheral tissue, IR appears earlier in the central nervous system, indicating that brain is more vulnerable to IR, especially under various pathological states such as ICH and ischemic stroke (IS) [5]. Therefore, the indicators associated with IR may be closely related to the initiation of ICH and its prognosis.

The triglyceride-glucose (TyG) index, consisting of fasting triglyceride (FTG) and fasting blood glucose (FBG), is a valuable tool for analyzing lipid and glucose metabolism [6, 7]. It is also recognized as an accurate indicator of IR [8, 9]. Some researchers have observed a positive correlation between the TyG index and the incidence and mortality rates of progressed coronary artery disease [10, 11]. Additional studies have indicated that the TyG index may have the potential to forecast negative cardiovascular events in individuals with coronary artery disease [12]. Moreover, multiple studies have demonstrated the predictive ability of the TyG index for the onset and mortality of IS [13, 14]. These findings collectively highlight the association of the TyG index with cardiovascular and cerebrovascular diseases. However, the relationship between ICH and the TyG index, as well as the prognostic role of the TyG index in this condition, remains unexplored.

Therefore, the objective of this study is to evaluate the impact of the TyG index on the prognosis of critically ill patients with ICH, which may establish its potential utility as a risk stratification tool in ICH cases.

## Materials

### Data sources

Data used in this study were extracted from the Medical Information Mart for Intensive Care (MIMIC-IV version 2.2) and the eICU Collaborative Research Database (eICU-CRD) [15, 16]. MIMIC-IV consists of medical records between 2008 and 2019 from over 190,000 patients who were treated in various types of ICU of the Beth Israel Deaconess Medical Center. The eICU-CRD included medical records of over 200,000 patients receiving clinical management in ICUs from over 200 medical centers between 2014 and 2015. Since data in these two databases are de-identified to hide patients’ information, the informed consent and ethics approval are not essential.

### Data extraction

Structure query language (SQL), executed on the PostgresSQL (version 13.7.2), was utilized for data extraction from MIMIC-IV and eICU-CRD. One researcher (Yang Yang) with authorization to access both databases (Record ID: 48,776,647) conducted the data extraction. Inclusive criteria encompassed patients who were (1) aged 18 years and above; (2) diagnosed with ICH in accordance with International Classification of Diseases, 9th and 10th Revision (ICD9 and ICD10). Exclusion criteria included: (1) patients with multiple hospitalization entries, only data from the initial hospitalization due to ICH were considered; (2) patients lacking data of FTG and FBG on the first day of ICU admittance were omitted; (3) individuals who expired or were released within 24 h of ICU admission were excluded due to their significant missing data for key variables used in the regression analysis. Therefore, excluding this group of patients was necessary to ensure the reliability of the results of the regression analysis.

The following information was extracted for the final study cohort: (1) patients’ age and gender; (2) comorbidities identified by ICD-9 and ICD-10 codes; (3) initial FBG and FTG results within 24 h post-ICU admission; (4) average values of laboratory parameters within 24 h of ICU admittance; (5) minimum Glasgow Coma Scale (GCS) score on the first day of ICU admittance; (6) maximum Acute Physiology Score III (APSIII) and Sequential Organ Failure Assessment (SOFA) scores on the first day of ICU management; (7) treatment-related data that may impact the prognosis of ICH patients were extracted, which includes invasive mechanical ventilation, the use of anticoagulants, and the use of antiplatelet agents during hospitalization.

### Assessment of the TyG index

**Fig. 1:**
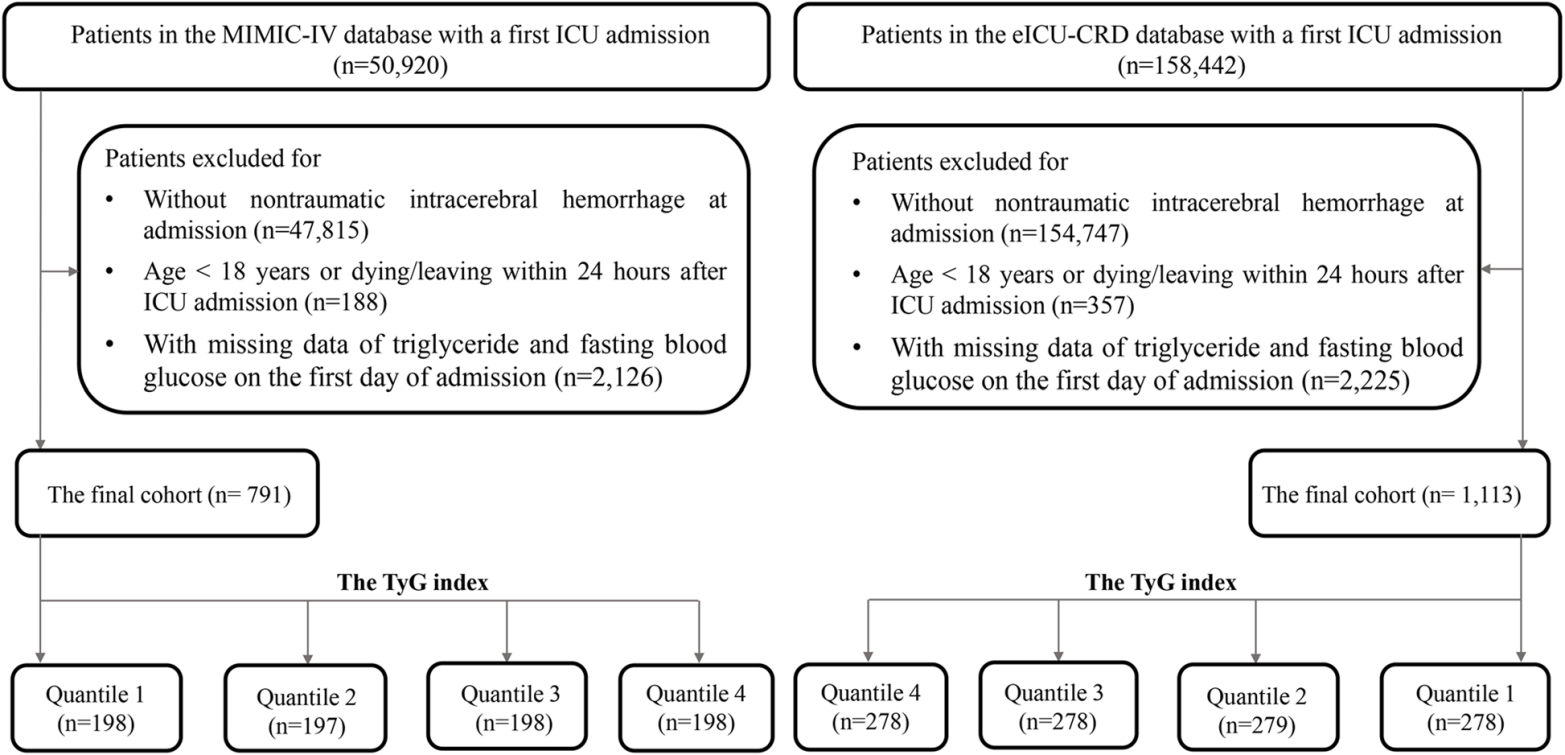
The flow chart for extracting data from the MIMIC-IV and eICU databases

The TyG index is calculated using the formula: TyG index = ln [FTG (mg/dl) × FBG (mg/dl)/2], where FTG and FBG represent the first recorded values of FBG and FTG since ICU admission [17, 18]. In the subsequent statistical analysis, the TyG index was considered both as a continuous and categorical variable. When treated as a categorical variable, it was divided into four groups based on quartiles. The data extraction process is illustrated in Fig. 1.

### Primary outcomes

The primary outcomes of interest were all-cause in-hospital mortality and ICU mortality, which were defined as deaths occurring during hospitalization and ICU staying, respectively.

### Statistical analysis

Continuous variables were expressed as median (interquartile ranges) and categorical variables were described as number (percentages). Comparisons between groups were performed by Mann–Whitney U or Kruskal–Wallis test for continuous variables, and chi-squared or Fisher’s exact test for categorical ones.

In order to investigate the relationship between the TyG index and the primary outcomes, an initial analysis utilizing restricted cubic splines (RCS) with four knots was carried out to assess any potential non-linear associations between the TyG index and the risk of in-hospital and ICU mortality. If a non-linear relationship was not detected, logistic regression analysis were performed using three different models: model 1 included only the TyG index, model 2 adjusted for age and gender, and model 3 further adjusted for various potential confounders relevant to the clinical outcomes of ICH, including GCS, hypertension, congestive heart failure, white blood cell count (WBC), serum urea nitrogen (BUN), serum creatinine, red cell distribution width (RDW), serum bilirubin, serum aspartate aminotransferase (AST), prothrombin time (PT), use of anticoagulants, and use of antiplatelet agents. Additionally, to check for multicollinearity in the logistic regression analysis, a Spearman rank correlation test was carried out and the square root of the variance inflation factor (VIF) was calculated.

To explore potential variations within specific populations, subgroup analysis was conducted by stratifying patients according to gender, age (> 60 vs. ≤ 60 years), diabetes, hypertension, use of anticoagulants, and use of antiplatelet agents. The interaction between the TyG index and the other variables utilized for stratification in subgroup analysis was evaluated through likelihood ratio test. Finally, a sensitivity analysis was performed by using Cox proportional hazard regression to verify the relationship between the TyG index and in-hospital and ICU mortality. The follow-up period was measured from the date of hospital or ICU admission to the date of death during the hospitalization or ICU stay. The Cox regression model was adjusted for possible confounders as outlined in the fully adjusted model (model 3) of logistic regression mentioned above.

All statistical analysis were performed using R software (version 4.3.1). The“VIM”package was used to visualize the distribution of missing values, from which we can see that all variables had missing ratio less than 20% (Additional file 1: Figure S1). The “mice” package was adopted to address missing values by multiple imputation to obtain 5 imputation datasets in the process of logistic regression. Besides, the “corrplot” package was used to visualize the associations between continuous variables. The“plotRCS” package was used to perform RCS. The“forestploter” package was adopted to visualized the results of subgroup analysis. The “survminer” package was used to conduct Cox regression analysis. Statistically significant was set as a two tailed *P* < *0.05*.

## Results

### Baseline characteristics

A total of 791 patients from MIMIC-IV and 1,113 from eICU-CRD were included in the final analysis. Among them, 418 (53%) individuals in MIMIC-IV and 627 (56%) in eICU-CRD were male. The in-hospital mortality rates were 14% in MIMIC-IV and 16% in eICU-CRD, with ICU mortality rates of 10% and 8% respectively. The median age was 72.25 (60.63, 82.59) years in MIMIC-IV and 66 (55, 77) years in eICU-CRD. Besides, the average value of TyG index was 8.72 (8.38, 9.17) in MIMIC-IV and 8.76 (8.33, 9.21) in eICU-CRD.

When dividing participants into groups based on the quartiles of the TyG index, patients in the upper quartiles had significantly higher APSIII scores, and higher proportion of invasive ventilation than those in the lower quartiles (*P* < *0.001 for all*). Furthermore, hospital stay time, ICU stay time, in-hospital mortality, and ICU mortality all exhibited a gradual increase from the first to the fourth quartile of the TyG index. However, there was no significant difference in the mean hospital and ICU survival time of patients who died in the hospital or ICU across the quartiles of the TyG index. (Table 1 and Additional file 2: Table S1).

**Table 1:** Baseline characteristics of participants from MIMIC-IV grouped by TyG index quartiles ^a^^,b^

Variables	Total (*n* = 791)	Q1 (*n* = 198)	Q2 (*n* = 197)	Q3 (*n* = 198)	Q4 (*n* = 198)	*P* value
Age, years	72.25 (60.63, 82.59)	78.24 (64.55, 86.05)	75.05 (64.73, 83.57)	71.78 (60.61, 81.08)	64.69 (53.77, 76.22)	< 0.001
Male, n%	418 (53)	88 (44)	118 (60)	97 (49)	115 (58)	0.005
GCS	14 (12, 15)	13 (11, 14)	14 (11, 15)	14 (12, 15)	15 (12, 15)	< 0.001
**Severe Score**						
APSIII	34 (26, 43.5)	33 (25, 43)	33 (25, 42)	33.5 (26, 44)	37 (27, 48)	0.024
SOFA	3 (1, 4)	3 (1, 4)	2 (1, 4)	3 (1, 4)	3 (2, 5)	0.048
**Comorbidities, n(%)**						
MI	60 (8)	16 (8)	10 (5)	16 (8)	18 (9)	0.467
CHF	76 (10)	27 (14)	15 (8)	17 (9)	17 (9)	0.159
Diabetes	185 (23)	45 (23)	42 (21)	53 (27)	45 (23)	0.609
Renal.disease	72 (9)	25 (13)	15 (8)	15 (8)	17 (9)	0.249
PVD	38 (5)	15 (8)	8 (4)	10 (5)	5 (3)	0.120
COPD	79 (10)	23 (12)	15 (8)	18 (9)	23 (12)	0.461
Hypertension	516 (65)	127 (64)	136 (69)	132 (67)	121 (61)	0.388
**Laboratory test**						
WBC (K/uL)	9.85 (7.81, 12.67)	8.9 (7.3, 11.19)	9.82 (7.54, 11.8)	10.25 (8.35, 13.22)	10.57 (8.36, 14)	< 0.001
Hemoglobin (g/dL)	12.7 (11.5, 13.8)	12.38 (11.22, 13.4)	12.8 (11.75, 14.1)	12.78 (11.66, 13.84)	12.7 (11.3, 13.8)	0.019
Platelets (K/uL)	211 (169, 258)	203.5 (166.12, 249.75)	199 (162.25, 250.25)	218 (178.25, 266.25)	220 (172.75, 263.88)	0.194
RDW (%)	13.6 (13, 14.55)	13.6 (12.95, 14.4)	13.5 (13, 14.4)	13.72 (13.1, 14.6)	13.7 (13, 14.7)	0.409
Calcium (mg/dL)	8.85 (8.44, 9.2)	8.8 (8.55, 9.2)	8.9 (8.5, 9.25)	8.85 (8.4, 9.2)	8.75 (8.3, 9.2)	0.289
Sodium (mEq/L)	139.5 (137.5, 142)	139.5 (138, 142)	140 (138, 142)	140 (137.5, 142.5)	139.5 (137.5, 142)	0.828
Potassium (mEq/L)	4 (3.7, 4.3)	3.95 (3.7, 4.14)	3.9 (3.7, 4.2)	4 (3.65, 4.3)	4.07 (3.75, 4.34)	0.015
Creatinine (mg/dL)	0.9 (0.75, 1.1)	0.8 (0.7, 0.95)	0.9 (0.75, 1.1)	0.95 (0.8, 1.15)	1 (0.8, 1.34)	< 0.001
BUN (mg/dL)	16.5 (12.5, 21.75)	15 (12, 19.5)	16.5 (12.5, 21)	17.5 (13, 22)	17.5 (13, 24.88)	0.002
Bilirubin (mg/dL)	0.6 (0.4, 0.8)	0.6 (0.4, 0.8)	0.65 (0.5, 0.9)	0.6 (0.4, 0.8)	0.6 (0.4, 0.8)	0.187
ALT^c^	1.28 (1.15, 1.45)	1.22 (1.11, 1.34)	1.36 (1.18, 1.48)	1.26 (1.15, 1.41)	1.32 (1.2, 1.53)	< 0.001
AST^d^	1.41 (1.29, 1.57)	1.37 (1.26, 1.49)	1.45 (1.32, 1.61)	1.38 (1.26, 1.55)	1.44 (1.3, 1.64)	< 0.001
PT (s)	12.4 (11.5, 13.8)	12.1 (11.5, 13.74)	12.55 (11.7, 14.1)	12.3 (11.4, 13.59)	12.5 (11.55, 13.8)	0.148
APTT (s)	27.85 (25.4, 30.85)	28 (25.85, 30.65)	28.42 (25.49, 31.31)	27.4 (25.45, 30.65)	27.48 (24.81, 30.88)	0.287
TG (mg/dL)	95 (71, 137)	62 (51, 71)	86 (74, 98)	117 (99.25, 138.75)	173.5 (134.25, 250.5)	< 0.001
FBG (mg/dL)	124 (106.5, 151)	109 (97, 125.38)	120.5 (106, 139)	127 (108.62, 150)	161.5 (126.5, 213.12)	< 0.001
TyG index	8.72 (8.38, 9.17)	8.14 (7.99, 8.27)	8.56 (8.47, 8.63)	8.92 (8.83, 9.03)	9.49 (9.29, 9.86)	< 0.001
**Events**						
ICU-stay time (day)	3.08 (1.72, 6.89)	2.64 (1.48, 4.79)	3.37 (1.78, 7.48)	2.91 (1.53, 6.64)	4.39 (2.11, 9.99)	< 0.001
Hospital-stay time (day)	7.06 (4.00, 13.66)	6.62 (4.01, 11.05)	7.6 (4.05, 13.82)	6.69 (3.72, 12.74)	8.89 (4.66, 17.62)	0.016
ICU-survive time (day)^e^	4.48 (2.78, 8.28)	4.31 (2.77, 8.18)	4.22 (2.07, 8.49)	3.8 (2.6, 7.12)	4.9 (3, 9.59)	0.936
Hospital-survive time (day)^f^	5.43 (3.12, 11.68)	5.61 (3.28, 11.22)	6.32 (3.19, 14.04)	4.27 (3.02, 7.85)	5.69 (3.08, 11.68)	0.712
Hospital mortality (%)	110 (14)	18 (9)	26 (13)	24 (12)	42 (21)	0.004
ICU mortality (%)	78 (10)	13 (7)	15 (8)	17 (9)	33 (17)	0.003
**Medication**						
Invasive ventilation (%)	275 (35)	37 (19)	63 (32)	71 (36)	104 (53)	< 0.001
Statin agents (%)	356 (45)	83 (42)	85 (43)	90 (45)	98 (49)	0.446
Anticoagulant agents (%)	629 (80)	153 (77)	156 (79)	159 (80)	161 (81)	0.779
Antiplatelet agents (%)	277 (35)	58 (29)	73 (37)	64 (32)	82 (41)	0.060

*Abbreviations*: *GCS* Glasgow coma scale, *APSIII* acute physiology score III, *SOFA* Sequential Organ Failure Assessment, *MI* myocardial infarct, *CHF* congestive heart failure, *PVD* peripheral vascular disease, *CPOD* chronic obstructive pulmonary disease, *WBC* white blood cell count, *RDW* red cell distribution width, *BUN* blood urea nitrogen, *ALT* alanine aminotransferase, *AST* aspartate aminotransferase, *PT* prothrombin time, *APTT* activated partial thromboplastin time, *TG* triglycerides, *FBG* fasting blood glucose, *TyG index* triglyceride glucose index

^a^Continuous data is presented as median (interquartile range), whereas categorical data are presented as frequency (percentage)

^b^TyG index: Q1 (7.29–8.38), Q2 (8.38–8.72), Q3 (8.72–9.17), Q4 (9.17–12.08)

^c^ALT in the table is the value after logarithmic transformation

^d^AST in the table is the value after logarithmic transformation

^e^ICU-survive time represents the average survival time of ICU deceased patients in each the TyG index quartile

^f^Hospital-survive time represents the average survival time of in-hospital deceased patients in each the TyG index quartile

Baseline data of participants divided by the hospital and ICU outcomes are presented in Table 2 and Additional file 3: Table S2, respectively. Compared to in-hospital and ICU survivors, non-survivors in both the MIMIC-IV and eICU-CRD databases showed significantly higher APSIII and SOFA scores, shorter hospital stays, and a higher proportion of invasive ventilation. However, compared to in-hospital and ICU survivors, ICU stay time was shorter in non-survivors from eICU-CRD and longer in non-survivors from MIMIC-IV. Furthermore, GCS scores were lower in in-hospital and ICU non-survivors compared to survivors in eICU-CRD, but there was no significant difference in GCS scores between in-hospital and ICU survivors and non-survivors in MIMIC-IV. Interestingly, despite the potential risk of secondary hemorrhage associated with antiplatelet agents, their usage was more common among in-hospital and ICU non-survivors than survivors in MIMIC-IV. Moreover, the TyG index was notably higher in the in-hospital non-survivors compared to survivors (MIMIC-IV: 8.94 (8.51–9.48) vs. 8.70 (8.36–9.09); *P* < *0.001*. eICU-CRD: 8.98 (8.51–9.49) vs. 8.70 (8.31–9.16); *P* < *0.001*). Similarly, the TyG index was significantly elevated in ICU non-survivors in contrast to ICU survivors (MIMIC-IV: 9.00 (8.51–9.48) vs. 8.70 (8.36–9.10); *P* < 0.001. eICU-CRD: 9.09 (8.76–9.65) vs. 8.71 (8.31–9.17); *P* < 0.001) (Additional file 4: Figure S2).

**Table 2:** Baseline characteristics of the in-hospital survivors and non-survivors in MIMIC-IV and eICU-CRD databases^a^

Variables	MIMIC-IV				eICU-CRD			
	**Total (*n* = 791)**	**Survivors (*n* = 681)**	**Non-survivors (*n* = 110)**	***P* value**	**Total (*n* = 1113)**	**Survivors (*n* = 939)**	**Non-survivors (*n* = 174)**	***P* value**
Age, years	72.25 (60.63, 82.59)	71.44 (60.34, 82.29)	76.35 (66.01, 84.42)	0.023	66 (55, 77)	66 (54, 77)	68 (58.25, 79)	0.089
Male, n%	418 (53)	359 (53)	59 (54)	0.939	627 (56)	526 (56)	101 (58)	0.680
GCS	14 (12, 15)	14 (12, 15)	15 (10, 15)	0.089	13 (7, 14.5)	13 (9, 15)	5 (3, 9)	< 0.001
**Severe Score**								
APSIII	34 (26, 43.5)	32 (25, 41)	43.5 (34, 52)	< 0.001	34 (24, 51)	33 (23, 45)	57 (42, 80)	< 0.001
SOFA	3 (1, 4)	2 (1, 4)	4 (3, 6)	< 0.001	4 (3, 6)	4 (3, 6)	5 (3, 7)	0.009
**Comorbidities, n(%)**								
MI	60 (8)	55 (8)	5 (5)	0.270	80 (7)	70 (7)	10 (6)	0.521
CHF	76 (10)	67 (10)	9 (8)	0.709	101 (9)	83 (9)	18 (10)	0.623
Diabetes	185 (23)	160 (23)	25 (23)	0.956	275 (25)	237 (25)	38 (22)	0.390
Renal disease	72 (9)	64 (9)	8 (7)	0.589	80 (7)	69 (7)	11 (6)	0.748
PVD	38 (5)	33 (5)	5 (5)	0.998	42 (4)	32 (3)	10 (6)	0.204
COPD	79 (10)	69 (10)	10 (9)	0.868	103 (9)	85 (9)	18 (10)	0.691
Hypertension	516 (65)	439 (64)	77 (70)	0.306	692 (62)	584 (62)	108 (62)	0.987
**Laboratory test**								
WBC (K/uL)	9.85 (7.81, 12.67)	9.7 (7.7, 12.3)	11.62 (8.64, 14.81)	< 0.001	10.09 (7.7, 12.8)	9.75 (7.55, 12.5)	11.41 (9.2, 15.4)	< 0.001
Hemoglobin (g/dL)	12.7 (11.5, 13.8)	12.75 (11.6, 13.8)	12.1 (10.6, 13.4)	0.001	13 (11.75, 14.2)	13.1 (11.93, 14.35)	12.68 (11, 13.62)	< 0.001
Platelets (K/uL)	211 (169, 258)	215 (173.5, 259.75)	182.75 (138.5, 240.8)	< 0.001	213.25 (172, 262.62)	215 (174, 264)	198.5 (156.25, 253.12)	0.006
RDW (%)	13.6 (13, 14.55)	13.55 (13, 14.45)	14 (13.25, 15.1)	< 0.001	13.8 (13.15, 14.74)	13.75 (13.1, 14.65)	14.35 (13.45, 15.45)	< 0.001
Calcium (mg/dL)	8.85 (8.44, 9.2)	8.9 (8.5, 9.2)	8.57 (8.05, 9)	< 0.001	8.8 (8.4, 9.1)	8.8 (8.4, 9.15)	8.6 (8.3, 8.95)	0.002
Sodium (mEq/L)	139.5 (137.5, 142)	139.5 (137.5, 142)	140 (138, 143.5)	0.109	139 (137, 141.5)	139 (137, 141)	140 (137.5, 143)	< 0.001
Potassium (mEq/L)	4 (3.7, 4.3)	4 (3.7, 4.25)	4 (3.76, 4.4)	0.245	3.85 (3.6, 4.1)	3.85 (3.6, 4.1)	3.8 (3.55, 4.1)	0.231
Creatinine (mg/dL)	0.9 (0.75, 1.1)	0.9 (0.75, 1.1)	1 (0.76, 1.62)	< 0.001	0.88 (0.69, 1.15)	0.87 (0.68, 1.1)	0.96 (0.7, 1.44)	0.001
BUN (mg/dL)	16.5 (12.5, 21.75)	16 (12.5, 21)	19.5 (15, 30.75)	< 0.001	15.5 (11, 21)	15 (11, 20.5)	17.5 (12, 25.25)	< 0.001
Bilirubin (mg/dL)	0.6 (0.4, 0.8)	0.6 (0.4, 0.8)	0.7 (0.58, 1.05)	< 0.001	0.6 (0.4, 0.9)	0.6 (0.4, 0.9)	0.65 (0.45, 1.05)	0.058
ALT^b^	1.28 (1.15, 1.45)	1.28 (1.15, 1.43)	1.34 (1.2, 1.58)	0.006	1.38 (1.23, 1.56)	1.4 (1.26, 1.56)	1.35 (1.2, 1.52)	0.179
AST^c^	1.41 (1.29, 1.57)	1.4 (1.28, 1.54)	1.54 (1.38, 1.77)	< 0.001	1.38 (1.26, 1.56)	1.38 (1.26, 1.54)	1.46 (1.31, 1.6)	0.003
PT (s)	12.4 (11.5, 13.8)	12.3 (11.45, 13.6)	13.07 (12.1, 14.95)	< 0.001	13.2 (11.75, 14.4)	13.2 (11.8, 14.3)	13.5 (11.75, 15.25)	0.068
APTT (s)	27.85 (25.4, 30.85)	27.7 (25.4, 30.8)	28.4 (25.17, 31.09)	0.501	28.1 (25.55, 31.6)	28 (25.7, 31.15)	28.7 (24.8, 33.05)	0.767
TG (mg/dL)	95 (71, 137)	95 (71, 134)	101 (70.25, 146.75)	0.376	95 (69, 142)	94 (68, 138)	105 (71.5, 160)	0.040
FBG (mg/dL)	124 (106.5, 151)	121.5 (105, 146)	144.75 (121.1, 186.8)	< 0.001	129 (110, 160.5)	127 (108, 153.75)	152.75 (126.5, 191)	< 0.001
TyG index	8.72 (8.38, 9.17)	8.7 (8.36, 9.09)	8.94 (8.51, 9.48)	< 0.001	8.76 (8.33, 9.21)	8.7 (8.31, 9.16)	8.98 (8.51, 9.49)	< 0.001
**Events (days)**								
ICU-stay time	3.08 (1.72, 6.89)	2.96 (1.59, 6.54)	4.88 (2.54, 9.42)	< 0.001	2.92 (1.58, 6.88)	3 (1.62, 6.94)	2.52 (1.25, 5.53)	0.012
Hospital-stay time	7.06 (4, 13.66)	7.46 (4.46, 13.82)	5.43 (3.12, 11.68)	0.005	7 (3.88, 13.04)	7.13 (4.04, 13.67)	4.96 (2.9, 10.71)	< 0.001
**Medication, n(%)**								
Invasive ventilation	275 (35)	188 (28)	87 (79)	< 0.001	394 (35)	315 (34)	79 (45)	0.004
Statin agents	356 (45)	322 (47)	34 (31)	0.002	201 (18)	169 (18)	32 (18)	0.987
Anticoagulant agents	629 (80)	553 (81)	76 (69)	0.005	216 (19)	180 (19)	36 (21)	0.718
Antiplatelet agents	277 (35)	255 (37)	22 (20)	< 0.001	120 (11)	105 (11)	15 (9)	0.386

*Abbreviations: GCS* Glasgow coma scale, *APSIII* acute physiology score III, *SOFA* Sequential Organ Failure Assessment, *MI* myocardial infarct, *CHF* congestive heart failure, *PVD* peripheral vascular disease, *COPD* chronic obstructive pulmonary disease, *WBC* white blood cell count, *RDW* red cell distribution width, *BUN* blood urea nitrogen, *ALT* alanine aminotransferase, *AST* aspartate aminotransferase, *PT* prothrombin time, *APTT* activated partial thromboplastin time, *TG* triglycerides, *FBG* fasting blood glucose, *TyG index*, triglyceride glucose index

^a^Continuous data is presented as median (interquartile range), whereas categorical data are presented as frequency (percentage)

^b^ALT in the table is the value after logarithmic transformation

^c^AST in the table is the value after logarithmic transformation

### Association between the TyG index and the primary outcomes

**Fig. 2:**
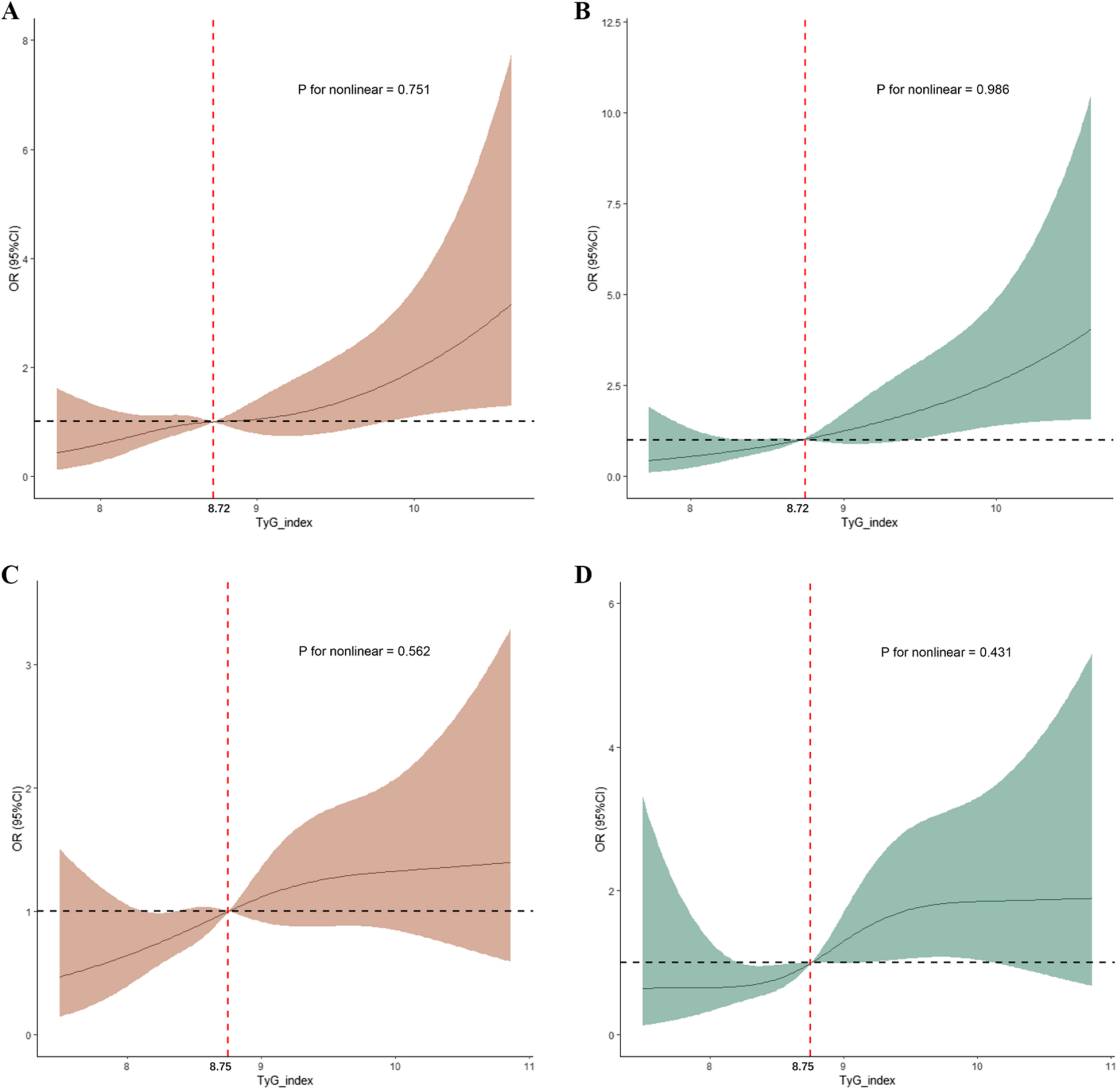
Restricted cubic spline analysis for the nonlinear association between the TyG index and the risk of, **A** in-hospital mortality of ICH patients from MIMIC-IV; **B** ICU mortality of ICH patients from MIMIC-IV; **C** in-hospital mortality of ICH patients from eICU-CRD; **D** ICU mortality of ICH patients from eICU-CRD

We initially conducted a nonlinear correlation analysis between the TyG index and the primary outcomes using RCS. Findings suggested no significant nonlinear correlation between the TyG indicator and the likelihood of either in-hospital or ICU mortality (In-hospital mortality: *P for nonlinear* = *0.751* in MIMIC-IV*, P for nonlinear* = *0.562* in eICU-CRD. ICU mortality: *P for nonlinear* = *0.986* in MIMIC-IV*, P for nonlinear* = *0.431* in eICU-CRD) (Fig. 2). Subsequently, logistic regression analysis was conducted to assess the linear relationship between the TyG index and the primary outcomes. In the fully adjusted model (model 3) that adjusted for various potential confounders related to the clinical outcomes of ICH, a positive correlation was found between the TyG index and the risk of in-hospital mortality (MIMIC-IV: OR 1.75 [95%CI 1.20–2.52], *P* = *0.003*. eICU-CRD: OR 1.37 [95%CI 1.05–1.80], *P* < *0.001*) and ICU mortality (MIMIC-IV: OR 2.15 [95%CI 1.45–3.17], *P* < *0.001.* eICU-CRD: OR 1.61 [95%CI 1.13–2.27], *P* < *0.001*). Moreover, compared to the first quartile (Q1) of the TyG index, the results of model 3 indicated that the fourth quartile (Q4) was linked to a higher risk of in-hospital mortality (MIMIC-IV: OR 2.31 [95%CI 1.18–4.67], *P* = *0.017*. eICU-CRD: 1.73 [95%CI 1.02–3.06], *P* = *0.036*) and ICU mortality (MIMIC-IV: OR 3.24 [95%CI 1.54–7.11], *P* = *0.002*. eICU-CRD: 2.30 [95%CI 1.09–5.16], *P* = *0.034*) (Table 3, Additional file 5: Table S3).

**Table 3:** The association between the TyG index and all cause in-hospital and ICU mortality in patients with ICH from MIMI-IV

Variables	Model 1			Model 2			Model 3		
	**OR(95% CI)**	***P *value**	***P-*trend**	**OR(95% CI)**	***P *value**	***P-*trend**	**OR(95% CI)**	***P *value**	***P-*trend**
**Hospital mortality**									
Continuous variable per unit	1.84 (1.37,2.48)	< 0.001		2.11 (1.55,2.90)	< 0.001		1.75 (1.20,2.52)	0.003	
Quantile^a^			< 0.001			< 0.001			0.017
Q1 (*n* = 198)	Ref								
Q2 (*n* = 197)	1.52 (0.81,2.91)	0.197		1.56 (0.82,3.00)	0.177		1.29 (0.65,2.60)	0.463	
Q3 (*n* = 198)	1.38 (0.73,2.66)	0.329		1.50 (0.79,2.91)	0.222		1.03 (0.51,2.11)	0.934	
Q4 (*n* = 198)	2.69 (1.51,4.97)	0.001		3.29 (1.80,6.22)	< 0.001		2.31 (1.18,4.67)	0.017	
**ICU mortality**									
Continuous variable per unit	1.96 (1.40,2.72)	< 0.001		2.15 (1.51,3.05)	< 0.001		2.15 (1.45,3.17)	< 0.001	
Quantile^a^			< 0.001			< 0.001			< 0.001
Q1 (*n* = 198)	Ref								
Q2 (*n* = 197)	1.17 (0.54,2.57)	0.685		1.18 (0.54,2.59)	0.679		1.07 (0.47,2.45)	0.872	
Q3 (*n* = 198)	1.34 (0.63,2.88)	0.449		1.42 (0.67,3.07)	0.366		1.30 (0.59,2.92)	0.524	
Q4 (*n* = 198)	2.85 (1.48,5.78)	0.002		3.26 (1.66,6.77)	< 0.001		3.24 (1.54,7.11)	0.002	

^a^TyG index: Q1 (7.29–8.38), Q2 (8.38–8.72), Q3 (8.72–9.17), Q4 (9.17–12.08)

Model 1: unadjusted

Model 2: adjusted for age and gender

Model 3: adjusted for age, gender, GCS, hypertension, congestive heart failure, WBC, serum creatinine, serum BUN, serum bilirubin, serum AST, PT, anticoagulant agents, and antiplatelet agents

To assess multicollinearity in the logistic regression model, the Spearman rank correlation coefficient and VIF were calculated, respectively. Findings revealed that there was no linear correlation between the TyG index and the other continuous variables incorporated in model 3 (Additional file 6: Figure S3). Additionally, none of the variables in model 3 exhibited a square root of VIF ≥ 2 (data not shown). Taken together, these results suggest that there is no multicollinearity present in the logistic regression model, indicating the reliability of the results.

### Subgroup analysis

**Fig. 3:**
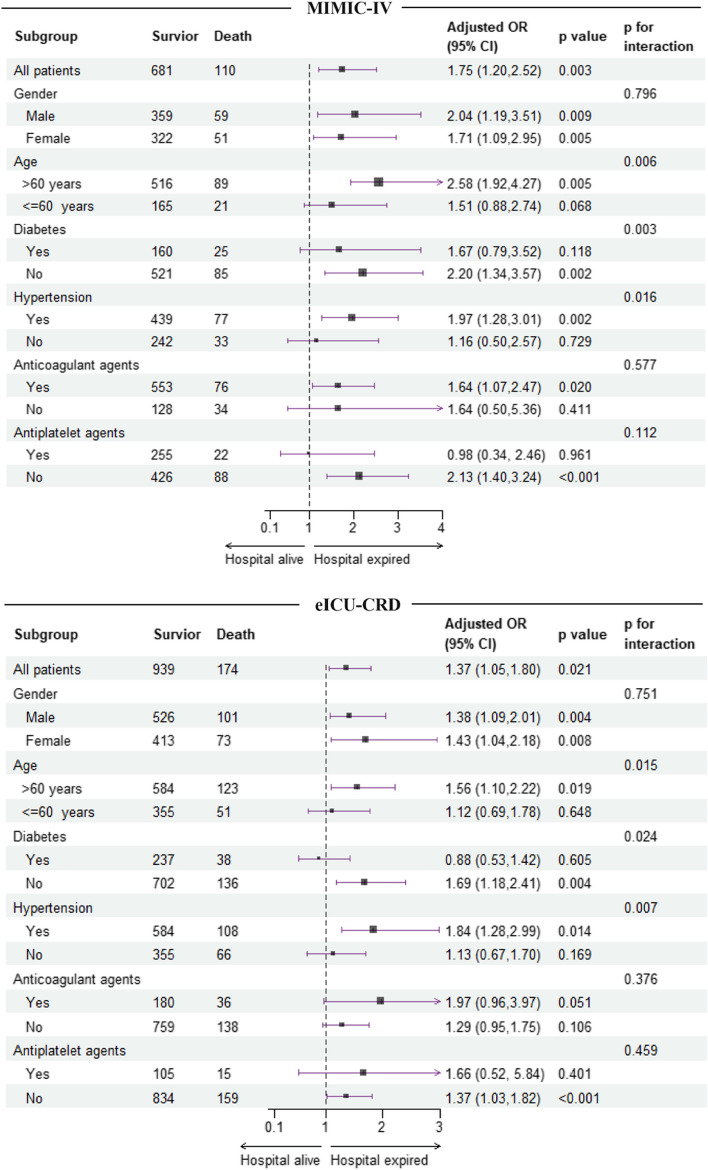
Subgroup analysis for the correlation between the TyG index and the risk of in-hospital mortality in patients with ICH from MIMIC-IV and eICU-CRD databases

**Fig. 4:**
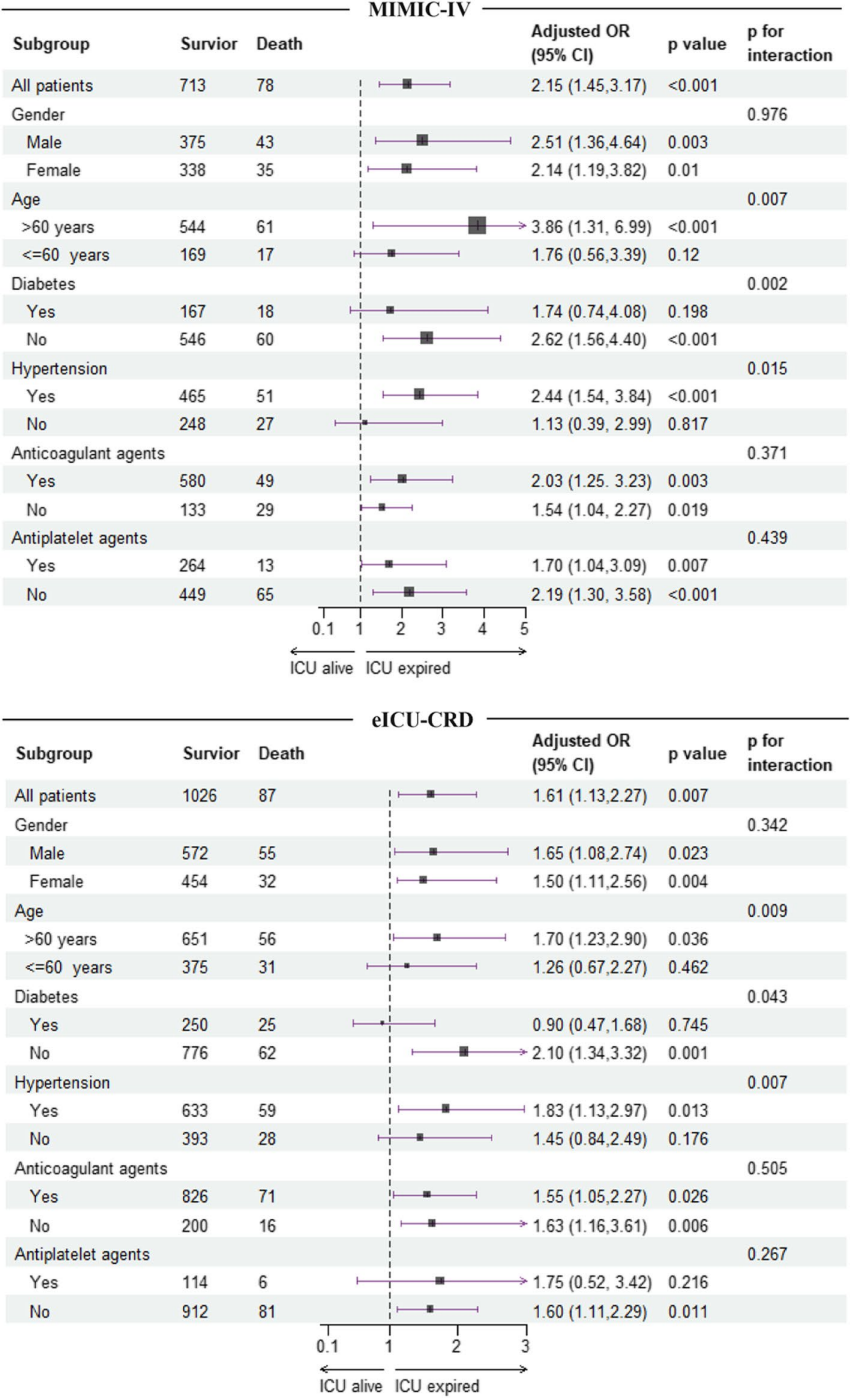
Subgroup analysis for the correlation between the TyG index and the risk of ICU mortality in patients with ICH from MIMIC-IV and eICU-CRD databases

To investigate potential variations within specific populations, logistic regression analysis was conducted across various subgroups, including gender, age, diabetes, hypertension, use of anticoagulant agents, and use of antiplatelet agents. The forest plot revealed a significant positive correlation between the TyG index and in-hospital mortality among participants over 60 years (MIMIC-IV: OR 2.58 [95%CI 1.92–4.27], *P* = *0.005*. eICU-CRD: OR 1.56 [95%CI 1.10–2.22], *P* = *0.019*), those without diabetes (MIMIC-IV: OR 2.20 [95%CI 1.34–3.57], *P* = *0.002*. eICU-CRD: OR 1.69 [95%CI 1.18–2.41], *P* = *0.004*), and those with hypertension (MIMIC-IV: OR 1.97 [95%CI 1.28–3.01], *P* = *0.002*. eICU-CRD: OR 1.84 [95%CI 1.28–2.99], *P* = *0.014*). Similarly, there was a positive association for ICU mortality in patients over 60 years (MIMIC-IV: OR 3.86 [95%CI 1.31–6.99], *P* < *0.001*. eICU-CRD: OR 1.70 [95%CI 1.23–2.90], *P* = *0.036*), those without diabetes (MIMIC-IV: OR 2.62 [95%CI 1.56–4.40], *P* < *0.001*. eICU-CRD: OR 2.10 [95%CI 1.34–3.32], *P* = *0.001*), and those with hypertension (MIMIC-IV: OR 2.44 [95%CI 1.54–3.84], *P* < *0.001*. eICU-CRD: OR 1.83 [95%CI 1.13–2.97], *P* = *0.013*). Furthermore, in both MIMIC-IV and eICU-CRD, significant interactions were found between the TyG index and patients' age, diabetic status, and history of hypertension concerning in-hospital and ICU outcomes of individuals with ICH (*P for interaction* < *0.05* for all) (Figs. 3 and 4).

### Sensitivity analysis

To further verify the association between the TyG index and in-hospital and ICU mortality, as well as the significant interactions between the TyG index and patients' age regarding in-hospital and ICU outcomes, a sensitivity analysis was performed using Cox proportional hazard regression. Following fully adjusted, a positive correlation was observed between the TyG index and the risk of in-hospital mortality in ICH patients from both MIMI-IV and eICU-CRD datasets. The positive association between the TyG index and in-hospital and ICU mortality was also present in patients over 60 years old, those without diabetes, and those with hypertension in both databases. Importantly, significant interactions were only found between patients' age and the TyG index concerning in-hospital and ICU outcomes of ICH patients in both MIMIC-IV and eICU-CRD datasets (Table 4 and Additional file 7: Table S4). These findings collectively suggest that the TyG index has the potential to serve as a prognostic indicator for ICH in patients over 60 years of age.

**Table 4:** Sensitivity analysis for the association between the TyG index and all cause in-hospital mortality^a,b^

Subgroup	MIMIC-IV			eICU-CRD		
	**HR(95% CI)**	***P value***	***P for*** ***interaction***	**HR(95% CI)**	***P value***	***P for*** ***interaction***
All patients	1.63 (1.20,2.22)	0.002		1.27 (1.04,1.59)	0.038	
Age			< 0.001			< 0.001
> 60 years	2.38 (1.38,4.46)	0.016		1.49 (1.13,1.86)	0.029	
≤ 60 years	1.55 (0.88,2.41)	0.085		1.05 (0.70,1.58)	0.806	
Diabetes			0.002			0.008
Yes	1.64 (0.86, 2.82)	0.374		1.06 (0.78, 1.52)	0.066	
No	2.59 (1.06, 6.35)	0.005		1.48 (1.12, 2.66)	0.005	
Hypertension			0.039			0.520
Yes	2.17 (1.36,3.46)	0.001		1.32 (1.03,1.86)	0.008	
No	1.76 (0.92,3.36)	0.085		1.22 (0.90,1.65)	0.121	

^a^Sensitivity analysis was conducted by the Cox proportional regression model

^b^Cox regression model was adjusted for age, gender, GCS, hypertension, congestive heart failure, WBC, serum creatinine, serum BUN, serum bilirubin, serum AST, PT, anticoagulant agents, and antiplatelet agents

## Discussion

In this retrospective multicenter study, the impact of the TyG index on the prognosis of critically ill patients with ICH was evaluated, uncovering two important findings. Firstly, a positive correlation was found between the TyG index and the risk of in-hospital and ICU all-cause mortality in ICH patients. Secondly, this correlation was notably stronger in patients over 60 years old, especially in those with hypertension or lacking diabetes.

The association between the TyG index and the course of IS has been extensively studied. Wang et al. reported that individuals in the highest quartile of the TyG index face a 1.45 times greater risk of developing IS compared to those in the lowest quartile [19]. Results from a 9-year prospective study showed that keeping TyG index elevated was strongly related to an increased morbidity of IS, suggesting that monitoring and regulating the TyG index at an appropriate level could be beneficial in preventing IS [20]. Additionally, various studies have explored the capability of the TyG index in predicting the outcome of IS. Lee and colleagues found that the TyG index could forecast an adverse functional outcome three months post-reperfusion in IS patients [21]. Yang et al. noted a correlation between higher TyG index and elevated rates of both recurrence and mortality within one year following an IS event [22]. In critically ill patients, Cai W et al. found that the TyG index may assist in identifying IS patients at high risk of all-cause mortality [14]. Despite these findings highlight the significant relationship between the TyG index and IS as well as its prognosis, research on the association between the TyG index and ICH remains scarce.

To address this gap in knowledge, we conducted this study and found a positive correlation between the TyG index and the likelihood of either in-hospital or ICU mortality in individuals with ICH. The positive correlation persisted even after adjusting for potential confounders, suggesting that the TyG index could serve as an independent predictor of hospitalization outcomes in patients with ICH, potentially aiding clinicians in their decision-making process. More importantly, subgroup analysis revealed that there is a synergistic effect of old age (over 60 years), hypertension, and non-diabetic status on the TyG index’s impact on hospitalization outcomes in patients with ICH. Sensitivity analysis using Cox regression model further confirmed the synergistic effect between old age (over 60 years) and the TyG index on the in-hospital and ICU mortality of ICH patients in both MIMIC-IV and eICU-CRD databases. These results underscored the population-specific influence of the TyG index on ICH prognosis, highlighting the importance of focusing on elderly patients with ICH.

The exact mechanisms connecting the TyG index with the poor prognosis of ICH are still unclear, but evidence supports a key role of IR in this process. Patients with IR are more susceptible to hyperglycemia. A study has found that hyperglycemia could inhibit the expression of Aquaporin-4, resulting in the aggravation of vasogenic brain edema and blood–brain barrier (BBB) destruction [23]. Autophagy is a vital cellular process for maintaining homeostasis, but hyperglycemia can decrease autophagic activity in the brain during ICH, leading to the accumulation of macromolecular debris and damaged cells, ultimately causing neuronal injury [24]. In stoke rat treated with type plasminogen activator, hyperglycemia could enhance superoxide production in brain tissue and blood vessels, increasing BBB permeability in the peri-ischemic area and leading to a 3- to fivefold rise in the volume of secondary hemorrhage after thrombolysis [25]. This finding establishes a link between hyperglycemia-induced superoxide production and the increased risk of hematoma expansion in IS. The generation of reactive oxygen species has been demonstrated during ICH [26, 27]. Moreover, studies have shown that an increase in blood glucose can exacerbate hematoma expansion in a rat model of ICH [28]. Therefore, it is plausible to infer that IR related hyperglycemia may result in poor outcomes of ICH by promoting superoxide production, thereby increasing the risk of hematoma expansion.

Adequate cerebral perfusion is crucial for determining the prognosis of patients with ICH. The automatic regulation ability of cerebral blood vessels plays a significant role in maintaining appropriate cerebral perfusion during cerebral hemorrhage. Studies have indicated that elevated intracranial pressure in patients with acute ICH can impair cerebrovascular autoregulation within a two-week period [29]. The myogenic response, which refers to the ability of smooth muscle cells to react to changes in blood pressure, is essential for preserving cerebrovascular autoregulation [30]. Animal studies have proved that IR can heighten the tension of cerebrovascular myogenic response, leading to a reduction in the diameter of the cerebrovascular lumen, subsequently causing brain tissue ischemia and nerve cell injury [31]. In addition, the smooth muscle activity of distal cerebral arteries is notably higher than that of proximal ones, making them more vulnerable to IR and resulting in impaired cerebral circulation function [30].

Chronic inflammatory response is one pathogenesis of IR, and IR in turn can reflect the level of systemic inflammation in the body [32]. Interestingly, our study revealed a gradual increase in WBC across quartile intervals of the TyG index, with values exceeding the upper limit of the reference range in the fourth quartile, suggesting an escalation of systemic inflammation during the course of ICH in patients with IR. Furthermore, the integrity of BBB was compromised during ICH, allowing the infiltration of peripheral immune cells and pro-inflammatory cytokines into the central nervous system. This heightened inflammatory response in the brain can further compromise the BBB, creating a detrimental cycle [33]. In addition, study has shown that dysregulation of the insulin signaling pathway can activate NF-κB, leading to the transcription and expression of inflammatory factors in the brain, thereby exacerbating neuroinflammation [34].

Intriguingly, although the evidence presented supports the significant role of IR in linking the TyG index to the unfavorable prognosis of intracerebral hemorrhage (ICH), our study found no correlation between the TyG index and either in-hospital or ICU mortality in ICH patients with a history of diabetes, a group known to have a higher risk of IR compared to non-diabetic individuals. Explaining the cause of this paradox is challenging. One potential reason could be reverse causality [14, 35], where patients diagnosed with diabetes may be more likely to accept appropriate treatment or adopt healthy lifestyle habits. This could lead to their analytical parameters being similar to or even lower than those of non-diabetic counterparts. Consistent to this theory, in our study, no significant differences were observed in FBG, FTC, and TyG indexes between diabetic and non-diabetic individuals in both the MIMC-IV and eICU-CRD study cohorts (TyG index: 8.71 (8.38, 9.17) vs 8.77 (8.39, 9.14), *P* = 0.673 for MIMIC-IV. 8.75 (8.34, 9.22) vs 8.78 (8.3, 9.17), *P* = 0.925 for eICU-CRD). Additionally, as diabetic patients may have adopted a healthier lifestyle than their non-diabetic counterparts, their prognosis might be improved in subgroup analyses stratified by diabetes status.

The hyper-insulinemic-euglycemic clamp is considered the most accurate method for detecting IR, but its practicality is limited due to high costs, time-consuming procedures, and invasiveness. The homeostasis model assessment index for IR (HOMA-IR) is commonly used in clinical settings to assess beta-cell function and detect IR [36]. Nevertheless, its applicability is restricted in patients undergoing insulin therapy or those with ineffective beta cells [37]. Besides, HOMA-IR relies on measuring insulin levels, which are not routinely checked in clinical practice. Therefore, researchers have introduced the TyG index as a potentially reliable and cost-effective alternative marker for IR. Using hyper-insulin-normoglycemia clamp technique as the gold standard, a study showed excellent predictive efficiency of the TyG index for IR, with sensitive and specificity of 96.5% and 85.0%, respectively [8]. David and colleagues also demonstrated that the TyG index outperforms FBG and FTG in diagnosing type 2 diabetes and monitoring its progression [38]. Given that FBG and FTG measurements are available in most healthcare facilities, the TyG index has the potential to be widely utilized in blood glucose management and could serve as a valuable tool for risk assessment in patients with ICH.

The study has several limitations. First, the location and volume of hemorrhage, which are crucial factors influencing the prognosis of ICH, could not be extracted from the databases. Therefore, future research should incorporate these indicators to further validate the current findings. Secondly, specific population such as the Chinese or African are scarce in the study cohort. Consequently, the conclusions derived from this study should be cautiously interpreted in these population. Thirdly, the impact of dynamic changes in the TyG index on the prognosis of ICH patients was not assessed in this study. Given that variability in the TyG index has been linked to the incidence of IS [20], further studies are needed to investigate the cumulative effect of the TyG index on the incidence and outcome of ICH. Fourthly, exclusion of patients without FTG and FBG data on the first day of ICU admission may introduce bias if the missing data pattern is not completely random. Last but not the least, the utilization of propofol, fibrate, and glucose, along with insulin infusion prior to hospitalization, could have a notable effect on FTG and FBG levels. Nevertheless, neither MIMIC-IV nor eICU-CRD databases contains information on pre-hospitalization medications. Therefore, further investigation is required to confirm the current findings by incorporating these treatment-related information before ICU admission.

## Conclusion

This study identified a positive correlation between the TyG index and in-hospital as well as ICU all-cause mortality in patients with ICH, particularly among individuals aged over 60 years with a history of hypertension. The findings indicate that the TyG index may be a useful tool for risk stratification in elderly patients with ICH, assisting clinicians in identifying high-risk individuals and providing timely intervention.

### Supplementary Information

**Additional file 1: Figure S1. **The proportion and distribution of missing data for variables in (A) MIMIC-IV database and (B) eICU-CRD database.

**Additional file 2. **

**Additional file 3. **

**Additional file 4: Figure S2.** The boxplot of the TyG index stratified by the in-hospital and ICU outcomes. (A) The level of the TyG index in hospital survivors and non-survivors from the MIMIC-IV database. (B) The level of the TyG index in hospital survivors and non-survivors from the eICU-CRD database. (C) The level of the TyG index in ICU survivors and non-survivors from the MIMIC-IV database. (D) The level of the TyG index in ICU survivors and non-survivors from the eICU-CRD database.

**Additional file 5. **

**Additional file 6: Figure S3.** The correlation between continuous variables in the cohort derived from (A) MIMIC-IV and (B) eICU-CRD.

**Additional file 7. **

## Data Availability

The available data for MIMIC-IV can be accessed from the website https://mimic.physionet.org/. The available data for eICU-CRD can be accessed from the website https://eicu-crd.mit.edu/. The datasets used and/or analyzed during the current study are available from the corresponding author on reasonable request.

## Abbreviations

APSIII: Acute Physiology Score III
AST: Serum aspartate aminotransferase
BBB: Blood–brain barrier
BUN: Serum urea nitrogen
eICU-CRD: The eICU Collaborative Research Database
FBG: Fasting blood glucose
FTG: Fasting triglyceride
GCS: Glasgow coma scale
HOMA-IR: The homeostasis model assessment index for IR
ICD: International Classification of Diseases
ICU: Intensive care unit
ICH: Intracerebral hemorrhage
IR: Insulin resistance
IS: Ischemic stroke
MIMIC: The Medical Information Mart for Intensive Care
OR: Odds ratios
PT: Prothrombin time
RCS: Restricted cubic spline
RDW: Red cell distribution width
SOFA: Sequential Organ Failure Assessment
SQL: Structure Query Language
TyG index: Triglyceride glucose index
VIF: Variance inflation factor
