# Supplementary figures and images for: Triglyceride-glucose index as a potential predictor for in-hospital mortality in critically ill patients with intracerebral hemorrhage: a multicenter, case–control study
Source: BMC Geriatr. 2024 May 1;24:385. doi: 10.1186/s12877-024-05002-4 (PMC11061935; doi:10.1186/s12877-024-05002-4)

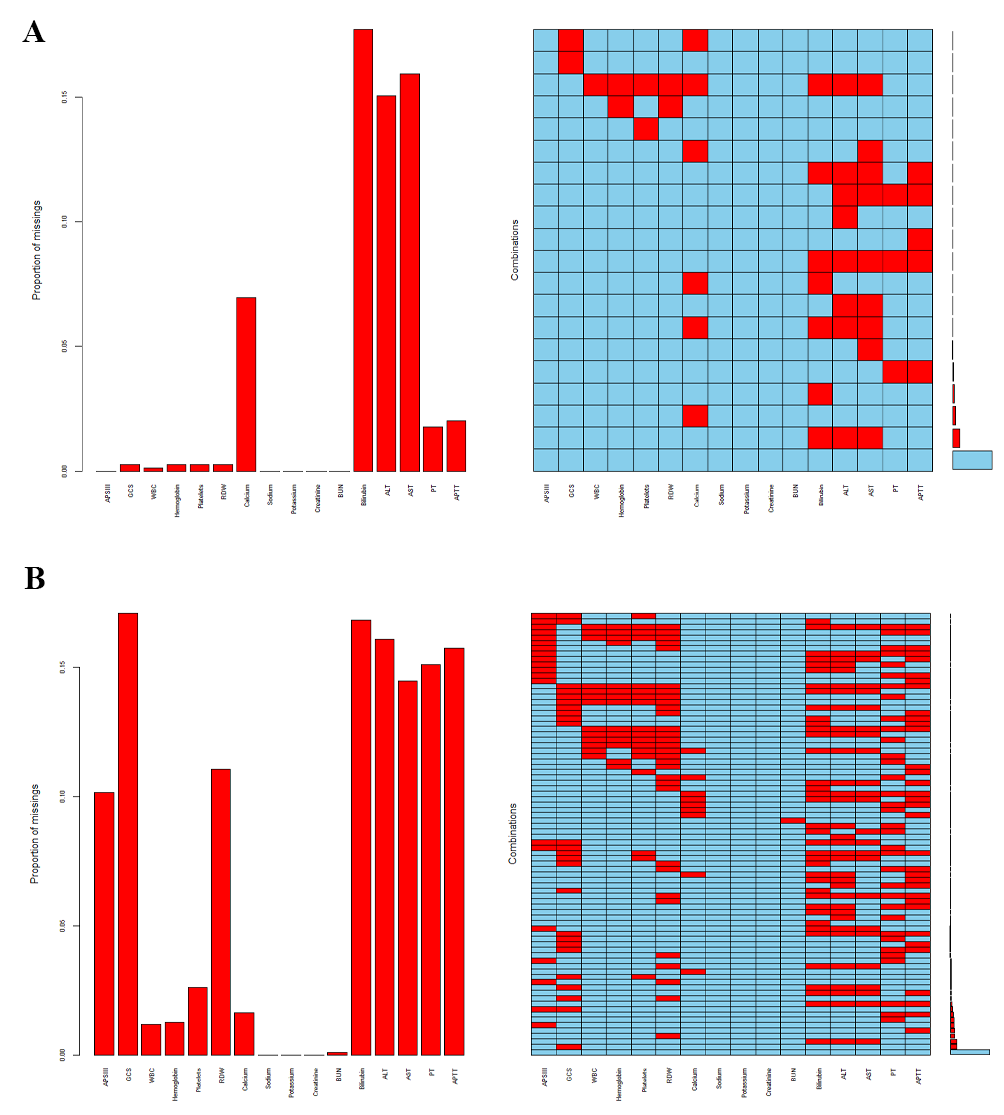

Supplement: Supplementary file 1 — Additional file 1: Figure S1. The proportion and distribution of missing data for variables in (A) MIMIC-IV database and (B) eICU-CRD database. [file 12877_2024_5002_MOESM1_ESM.tif]

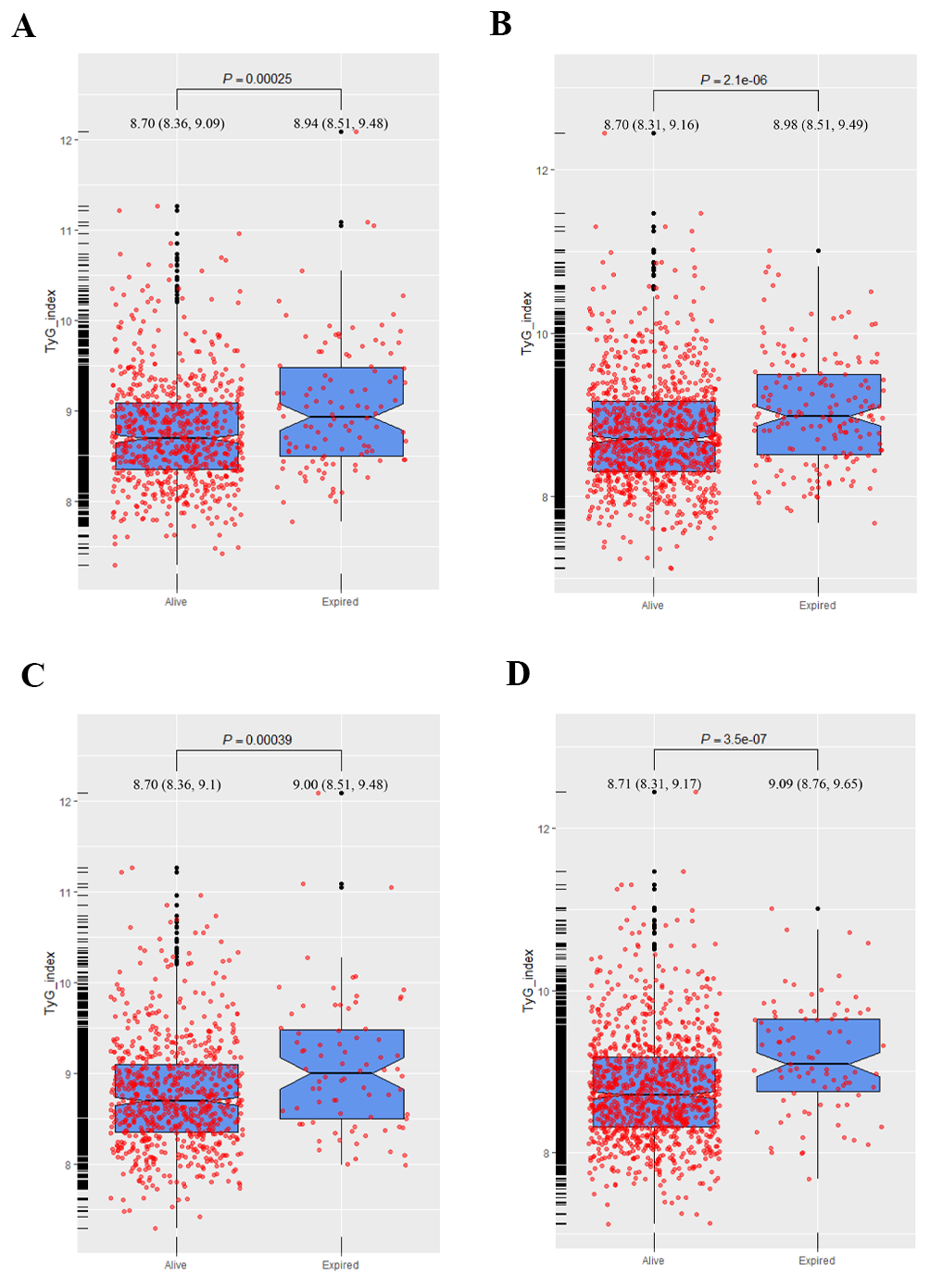

Supplement: Supplementary file 4 — Additional file 4: Figure S2. The boxplot of the TyG index stratified by the in-hospital and ICU outcomes. (A) The level of the TyG index in hospital survivors and non-survivors from the MIMIC-IV database. (B) The level of the TyG index in hospital survivors and non-survivors from the eICU-CRD database. (C) The level of the TyG index in ICU survivors and non-survivors from the MIMIC-IV database. (D) The level of the TyG index in ICU survivors and non-survivors from the eICU-CRD database. [file 12877_2024_5002_MOESM4_ESM.tif]

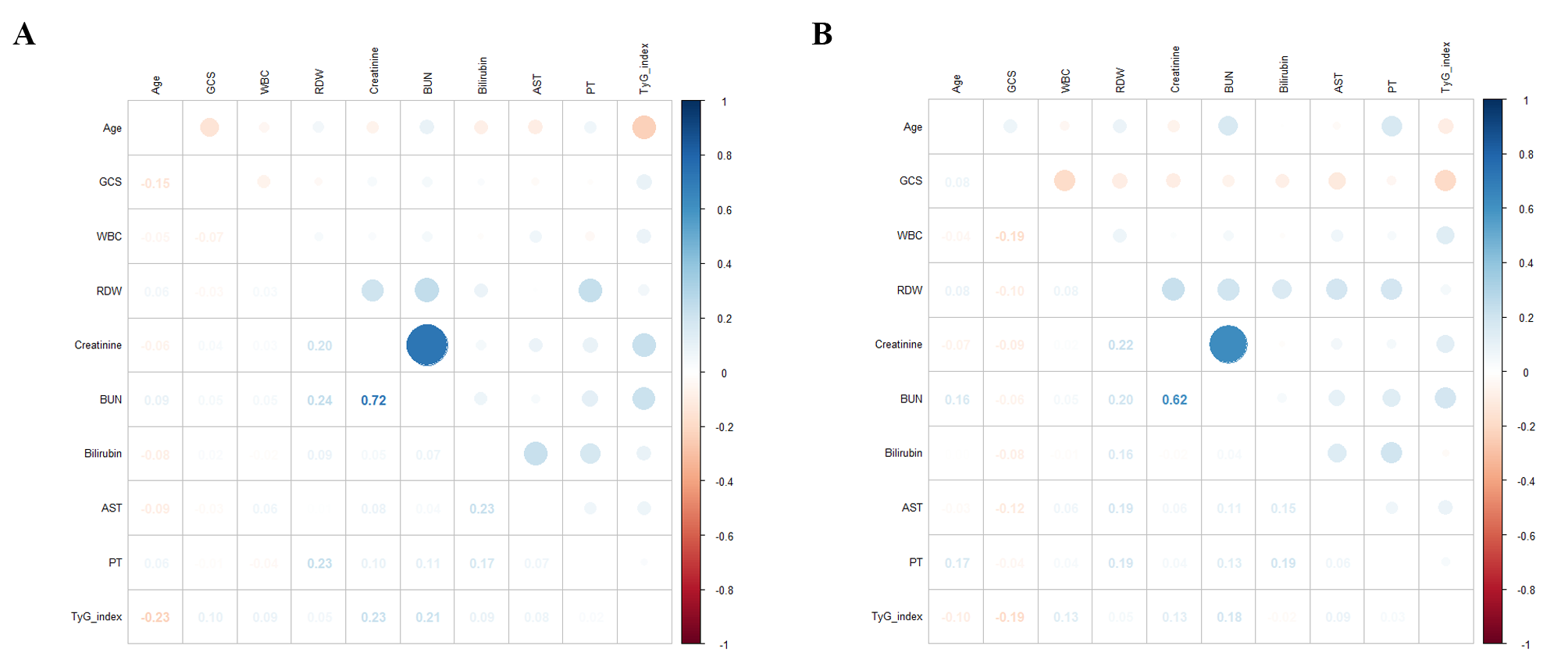

Supplement: Supplementary file 6 — Additional file 6: Figure S3. The correlation between continuous variables in the cohort derived from (A) MIMIC-IV and (B) eICU-CRD. [file 12877_2024_5002_MOESM6_ESM.tif]
