# Supplementary material for: Triglyceride-glucose index as a potential predictor for in-hospital mortality in critically ill patients with intracerebral hemorrhage: a multicenter, case–control study
Source: BMC Geriatr. 2024 May 1;24:385. doi: 10.1186/s12877-024-05002-4 (PMC11061935; doi:10.1186/s12877-024-05002-4)
Supplement: Supplementary file 2 — Additional file 2. [file 12877_2024_5002_MOESM2_ESM.docx]

**Table.S1 Baseline characteristics of participants from eICU-CRD grouped by TyG index quartiles ^a,b^**

| **Variables** | **Total  (n = 1113)** | **Q1  (n = 278)** | **Q2  (n = 279)** | **Q3  (n = 278)** | **Q4  (n = 278)** | ***P value*** |
| --- | --- | --- | --- | --- | --- | --- |
| Age, years | 66 (55, 77) | 67 (55, 78.75) | 68 (57, 78) | 68 (56.25, 78) | 62 (52, 71) | < 0.001 |
| Male, n% | 627 (56) | 149 (54) | 156 (56) | 166 (60) | 156 (56) | 0.540 |
| GCS | 13 (7, 14.5) | 14 (10, 15) | 13 (8, 15) | 12 (6.25, 14) | 10 (5, 14) | < 0.001 |
| **Severe Score** |  |  |  |  |  |  |
| APSIII | 34 (24, 51) | 30 (21, 41) | 33 (24, 45) | 35.5 (24, 50) | 45 (30, 67.5) | < 0.001 |
| SOFA | 4 (3, 6) | 4 (3, 6) | 4 (2, 6) | 4.5 (3, 7) | 5 (3, 7) | 0.175 |
| **Comorbidities, n (%)** |  |  |  |  |  |  |
| MI | 80 (7) | 23 (8) | 22 (8) | 19 (7) | 16 (6) | 0.659 |
| CHF | 101 (9) | 19 (7) | 33 (12) | 27 (10) | 22 (8) | 0.183 |
| Diabetes | 275 (25) | 72 (26) | 63 (23) | 75 (27) | 65 (23) | 0.588 |
| Renal.disease | 80 (7) | 20 (7) | 18 (6) | 31 (11) | 11 (4) | 0.011 |
| PVD | 42 (4) | 6 (2) | 12 (4) | 12 (4) | 12 (4) | 0.447 |
| COPD | 103 (9) | 25 (9) | 23 (8) | 30 (11) | 25 (9) | 0.761 |
| Hypertension | 692 (62) | 166 (60) | 166 (59) | 182 (65) | 178 (64) | 0.354 |
| **Laboratory test** |  |  |  |  |  |  |
| WBC (K/uL) | 10.09 (7.7, 12.8) | 8.6 (6.65, 11.4) | 9.8 (7.47, 12.15) | 10.32 (8.08, 13.1) | 11.62 (9.03, 14.7) | < 0.001 |
| Hemoglobin (g/dL) | 13 (11.75, 14.2) | 12.9 (11.9, 13.85) | 12.85 (11.65, 14.34) | 13.25 (11.7, 14.53) | 13.1 (11.72, 14.18) | 0.220 |
| Platelets (K/uL) | 213.25 (172, 262.62) | 207.25 (163.88, 253) | 214 (173, 262.75) | 210.5 (172, 256) | 231 (176.5, 279.12) | 0.003 |
| RDW (%) | 13.8 (13.15, 14.74) | 13.72 (13.1, 14.7) | 13.75 (13.2, 14.65) | 13.75 (13.1, 14.72) | 13.85 (13.2, 14.86) | 0.664 |
| Calcium (mg/dL) | 8.8 (8.4, 9.1) | 8.8 (8.46, 9.1) | 8.8 (8.45, 9.1) | 8.72 (8.3, 9.15) | 8.8 (8.35, 9.19) | 0.743 |
| Sodium (mEq/L) | 139 (137, 141.5) | 139.5 (137, 141) | 139 (137, 141.25) | 139 (137, 141.5) | 139 (136.62, 141) | 0.852 |
| Potassium (mEq/L) | 3.85 (3.6, 4.1) | 3.9 (3.65, 4.05) | 3.85 (3.6, 4.1) | 3.83 (3.55, 4.1) | 3.85 (3.6, 4.2) | 0.580 |
| Creatinine (mg/dL) | 0.88 (0.69, 1.15) | 0.78 (0.64, 0.99) | 0.86 (0.67, 1.04) | 0.94 (0.71, 1.2) | 1 (0.74, 1.33) | < 0.001 |
| BUN (mg/dL) | 15.5 (11, 21) | 13 (10, 18.5) | 15.5 (11, 20.5) | 16 (11.5, 22) | 17 (13, 24.5) | < 0.001 |
| Bilirubin (mg/dL) | 0.6 (0.4, 0.9) | 0.6 (0.4, 0.8) | 0.6 (0.4, 0.9) | 0.6 (0.45, 0.91) | 0.6 (0.4, 0.85) | 0.573 |
| ALT **^c^** | 1.38 (1.23, 1.56) | 1.38 (1.23, 1.52) | 1.34 (1.23, 1.53) | 1.38 (1.25, 1.56) | 1.44 (1.26, 1.6) | 0.104 |
| AST **^d^** | 1.38 (1.26, 1.56) | 1.36 (1.26, 1.56) | 1.37 (1.26, 1.53) | 1.38 (1.26, 1.52) | 1.45 (1.32, 1.63) | 0.004 |
| PT (s) | 13.2 (11.75, 14.4) | 13.1 (11.62, 14.2) | 13.1 (11.7, 14.3) | 13.35 (11.8, 14.75) | 13.25 (11.97, 14.25) | 0.468 |
| APTT (s) | 28.1 (25.55, 31.6) | 28.6 (26, 31) | 28.1 (25.5, 31.3) | 28.3 (25.45, 32.4) | 27.6 (25.02, 31.88) | 0.725 |
| TG (mg/dL) | 95 (69, 142) | 57 (47, 67.75) | 85 (72, 97) | 115.5 (95, 137) | 183 (143.25, 239.5) | < 0.001 |
| FBG (mg/dL) | 129 (110, 160.5) | 111.25 (95, 127) | 123 (106.5, 142) | 134.75 (116, 161.75) | 178.5 (135.12, 226.62) | < 0.001 |
| TyG index | 8.76 (8.33, 9.21) | 8.09 (7.92, 8.23) | 8.55 (8.44, 8.64) | 8.96 (8.85, 9.06) | 9.62 (9.38, 9.94) | < 0.001 |
| **Events** |  |  |  |  |  |  |
| ICU-stay time (day) | 2.92 (1.58, 6.88) | 2.62 (1.54, 4.94) | 3 (1.5, 7.38) | 3.04 (1.79, 7.17) | 3.17 (1.64, 7.58) | 0.027 |
| Hospital-stay time (day) | 7 (3.88, 13.04) | 6.73 (3.88, 11.66) | 6.75 (3.79, 11.84) | 7.69 (3.89, 12.94) | 7.8 (4.04, 16.3) | 0.024 |
| ICU-survive time (day)**^e^** | 2.33 (1.25, 3.88) | 1.5 (0.92, 2.81) | 3.12 (1.31, 6.9) | 2.62 (1.25, 5.71) | 2.31 (1.56, 3.23) | 0.598 |
| Hospital-survive time (day)**^f^** | 4.96 (2.9, 10.71) | 3.58 (2.62, 8.53) | 5.92 (2.54, 9.33) | 8.17 (2.88, 11.58) | 4.75 (3.07, 10.82) | 0.274 |
| Hospital mortality (%) | 174 (16) | 28 (10) | 29 (10) | 53 (19) | 64 (23) | < 0.001 |
| ICU mortality (%) | 87 (8) | 11 (4) | 11 (4) | 25 (9) | 40 (14) | < 0.001 |
| **Medication, n (%)** |  |  |  |  |  |  |
| Invasive ventilation | 394 (35) | 82 (29) | 83 (30) | 108 (39) | 121 (44) | < 0.001 |
| Statin agents | 201 (18) | 52 (19) | 51 (18) | 47 (17) | 51 (18) | 0.950 |
| Anticoagulant agents | 216 (19) | 58 (21) | 53 (19) | 58 (21) | 47 (17) | 0.594 |
| Antiplatelet agents | 120 (11) | 31 (11) | 26 (9) | 32 (12) | 31 (11) | 0.837 |

**^a^** *Continuous data is presented as median (interquartile range), whereas categorical data are presented as frequency (percentage).*

**^b^** *TyG index: Q1 (7.11–8.33), Q2 (8.33–8.76), Q3 (8.76–9.21), Q4 (9.21–12.44)*

**^c^** *ALT in the table is the value after logarithmic transformation.*

**^d^** *AST in the table is the value after logarithmic transformation.*

**^e^** *ICU-survive time represents the average survival time of ICU deceased patients in each the TyG index quartile.*

**^f^** *Hospital-survive time represents the average survival time of in-hospital deceased patients in each the TyG index quartile.*

*Abbreviation: GCS, Glasgow coma scale; APSIII, acute physiology score III;SOFA, Sequential Organ Failure Assessment;* *MI, myocardial infarct; CHF,* *congestive heart failure; PVD, peripheral vascular disease; CPOD, chronic obstructive pulmonary disease;* *WBC,* *white blood cell count; RDW,* *red cell distribution width; BUN,* *blood urea nitrogen; ALT, alanine aminotransferase; AST,* *aspartate aminotransferase; PT,* *prothrombin time; APTT, activated partial thromboplastin time; TG, triglycerides; FBG, fasting blood glucose; TyG index, triglyceride glucose index.*
