# Supplementary material for: Triglyceride-glucose index as a potential predictor for in-hospital mortality in critically ill patients with intracerebral hemorrhage: a multicenter, case–control study
Source: BMC Geriatr. 2024 May 1;24:385. doi: 10.1186/s12877-024-05002-4 (PMC11061935; doi:10.1186/s12877-024-05002-4)
Supplement: Supplementary file 3 — Additional file 3. [file 12877_2024_5002_MOESM3_ESM.docx]

**Table.S2 Baseline characteristics of the ICU survivors and non-survivors in MIMIC-IV and eICU-CRD databases ^a^**

| **Variables** | **MIMIC-IV** | | | |  | **eICU-CRD** | | | |
| --- | --- | --- | --- | --- | --- | --- | --- | --- | --- |
|  | **Total  (n = 791)** | **Survivors  (n = 681)** | **Non-survivors  (n = 110)** | ***P value*** |  | **Total  (n = 1113)** | **Survivors  (n = 939)** | **Non-survivors  (n = 174)** | ***P value*** |
| Age, years | 72.25 (60.63, 82.59) | 71.86 (60.59, 82.54) | 75.76 (62.46, 83.1) | 0.197 |  | 66 (55, 77) | 66 (55, 77) | 63 (53.5, 73.5) | 0.223 |
| Male, n% | 418 (53) | 375 (53) | 43 (55) | 0.76 |  | 627 (56) | 572 (56) | 55 (63) | 0.217 |
| GCS | 14 (12, 15) | 14 (12, 15) | 15 (9.25, 15) | 0.292 |  | 13 (7, 14.5) | 13 (8, 15) | 3 (3, 7) | < 0.001 |
| **Severe Score** |  |  |  |  |  |  |  |  |  |
| APSIII | 34 (26, 43.5) | 33 (25, 42) | 44 (37.25, 58.25) | < 0.001 |  | 34 (24, 51) | 33 (23, 47) | 67 (49, 89.5) | < 0.001 |
| SOFA | 3 (1, 4) | 3 (1, 4) | 5 (3, 6) | < 0.001 |  | 4 (3, 6) | 4 (3, 6) | 5 (3, 7) | 0.63 |
| **Comorbidities, n (%)** |  |  |  |  |  |  |  |  |  |
| MI | 60 (8) | 55 (8) | 5 (6) | 0.851 |  | 80 (7) | 77 (8) | 3 (3) | 0.234 |
| CHF | 76 (10) | 69 (10) | 7 (9) | 0.993 |  | 101 (9) | 97 (9) | 4 (5) | 0.187 |
| Diabetes | 185 (23) | 167 (23) | 18 (23) | 0.998 |  | 275 (25) | 250 (24) | 25 (29) | 0.437 |
| Renal disease | 72 (9) | 68 (10) | 4 (5) | 0.281 |  | 80 (7) | 76 (7) | 4 (5) | 0.448 |
| PVD | 38 (5) | 36 (5) | 2 (3) | 0.573 |  | 42 (4) | 38 (4) | 4 (5) | 0.564 |
| COPD | 79 (10) | 73 (10) | 6 (8) | 0.608 |  | 103 (9) | 93 (9) | 10 (11) | 0.577 |
| Hypertension | 516 (65) | 465 (65) | 51 (65) | 0.982 |  | 692 (62) | 633 (62) | 59 (68) | 0.31 |
| **Laboratory test** |  |  |  |  |  |  |  |  |  |
| WBC (K/uL) | 9.85 (7.81, 12.67) | 9.75 (7.8, 12.4) | 11.3 (8.38, 14.44) | 0.004 |  | 10.09 (7.7, 12.8) | 9.85 (7.6, 12.6) | 11.95 (9.9, 15.65) | < 0.001 |
| Hemoglobin (g/dL) | 12.7 (11.5, 13.8) | 12.7 (11.55, 13.8) | 12.2 (10.45, 13.5) | 0.03 |  | 13 (11.75, 14.2) | 13 (11.8, 14.3) | 12.7 (10.85, 13.7) | 0.009 |
| Platelets (K/uL) | 211 (169, 258) | 213 (172, 259.5) | 187 (131.75, 240.75) | 0.002 |  | 213.25 (172, 262.62) | 213.5 (173, 262.5) | 209.5 (156.5, 263.5) | 0.292 |
| RDW (%) | 13.6 (13, 14.55) | 13.6 (13, 14.5) | 14.05 (13.35, 15.1) | 0.002 |  | 13.8 (13.15, 14.74) | 13.75 (13.1, 14.7) | 14.25 (13.29, 15.24) | 0.033 |
| Calcium (mg/dL) | 8.85 (8.44, 9.2) | 8.85 (8.5, 9.2) | 8.6 (8.05, 9.1) | 0.005 |  | 8.8 (8.4, 9.1) | 8.8 (8.4, 9.15) | 8.68 (8.35, 8.9) | 0.069 |
| Sodium (mEq/L) | 139.5 (137.5, 142) | 139.5 (137.5, 142) | 140 (138, 143.5) | 0.047 |  | 139 (137, 141.5) | 139 (137, 141) | 140 (137.25, 143.25) | 0.046 |
| Potassium (mEq/L) | 4 (3.7, 4.3) | 4 (3.7, 4.25) | 4.03 (3.8, 4.47) | 0.097 |  | 3.85 (3.6, 4.1) | 3.85 (3.6, 4.1) | 3.8 (3.55, 4.1) | 0.407 |
| Creatinine (mg/dL) | 0.9 (0.75, 1.1) | 0.9 (0.75, 1.1) | 1 (0.76, 1.69) | 0.006 |  | 0.88 (0.69, 1.15) | 0.88 (0.69, 1.12) | 1.02 (0.72, 1.46) | 0.013 |
| BUN (mg/dL) | 16.5 (12.5, 21.75) | 16 (12.5, 21.5) | 19.25 (15.62, 28.75) | < 0.001 |  | 15.5 (11, 21) | 15.5 (11, 21) | 17 (12.25, 27.5) | 0.027 |
| Bilirubin (mg/dL) | 0.6 (0.4, 0.8) | 0.6 (0.4, 0.8) | 0.75 (0.6, 1.2) | < 0.001 |  | 0.6 (0.4, 0.9) | 0.6 (0.4, 0.9) | 0.65 (0.4, 1.02) | 0.561 |
| ALT **^b^** | 1.34 (1.2, 1.51) | 1.34 (1.2, 1.51) | 1.38 (1.2, 1.63) | 0.068 |  | 1.38 (1.23, 1.56) | 1.38 (1.24, 1.56) | 1.4 (1.2, 1.65) | 0.733 |
| AST **^c^** | 1.4 (1.28, 1.57) | 1.38 (1.28, 1.54) | 1.51 (1.37, 1.76) | < 0.001 |  | 1.38 (1.26, 1.56) | 1.38 (1.26, 1.54) | 1.49 (1.3, 1.67) | 0.004 |
| PT (s) | 12.4 (11.5, 13.8) | 12.35 (11.5, 13.65) | 13.07 (12.14, 15.47) | < 0.001 |  | 13.2 (11.75, 14.4) | 13.2 (11.8, 14.3) | 13.28 (11.74, 14.88) | 0.345 |
| APTT (s) | 27.85 (25.4, 30.85) | 27.7 (25.4, 30.77) | 29.45 (24.79, 31.83) | 0.294 |  | 28.1 (25.55, 31.6) | 28 (25.55, 31.3) | 29.2 (25.25, 33) | 0.225 |
| TG (mg/dL) | 95 (71, 137) | 95 (72, 135) | 101 (67.25, 147) | 0.529 |  | 95 (69, 142) | 94 (68, 137.75) | 114 (80, 179.5) | 0.001 |
| FBG (mg/dL) | 124 (106.5, 151) | 122 (105.5, 146.5) | 153 (124, 191.62) | < 0.001 |  | 129 (110, 160.5) | 128 (109, 156.38) | 158 (130.25, 201) | < 0.001 |
| TyG index | 8.72 (8.38, 9.17) | 8.7 (8.36, 9.1) | 9 (8.51, 9.48) | < 0.001 |  | 8.76 (8.33, 9.21) | 8.71 (8.31, 9.17) | 9.09 (8.76, 9.65) | < 0.001 |
| **Events (days)** |  |  |  |  |  |  |  |  |  |
| ICU-stay time | 3.08 (1.72, 6.89) | 3 (1.62, 6.66) | 4.48 (2.78, 8.28) | < 0.001 |  | 2.92 (1.58, 6.88) | 2.98 (1.62, 6.92) | 2.33 (1.25, 3.88) | 0.012 |
| Hospital-stay time | 7.06 (4, 13.66) | 7.61 (4.52, 13.99) | 3.89 (2.68, 8.22) | < 0.001 |  | 7 (3.88, 13.04) | 7.21 (4, 13.62) | 3.83 (2.56, 8.36) | < 0.001 |
| **Medication, n (%)** |  |  |  |  |  |  |  |  |  |
| Invasive ventilation | 275 (35) | 210 (29) | 65 (83) | < 0.001 |  | 394 (35) | 354 (35) | 40 (46) | 0.042 |
| Statin agents | 356 (45) | 335 (47) | 21 (27) | 0.001 |  | 201 (18) | 192 (19) | 9 (10) | 0.071 |
| Anticoagulant agents | 629 (80) | 580 (81) | 49 (63) | < 0.001 |  | 216 (19) | 200 (19) | 16 (18) | 0.914 |
| Antiplatelet agents | 277 (35) | 264 (37) | 13 (17) | < 0.001 |  | 120 (11) | 114 (11) | 6 (7) | 0.3 |

**^a^** *Continuous data is presented as median (interquartile range), whereas categorical data are presented as frequency (percentage).*

**^b^** *ALT in the table is the value after logarithmic transformation.*

**^c^** *AST in the table is the value after logarithmic transformation.*

*Abbreviation: GCS, Glasgow coma scale; APSIII, acute physiology score III; SOFA, Sequential Organ Failure Assessment;* *MI, myocardial infarct; CHF,* *congestive heart failure; PVD, peripheral vascular disease; COPD, chronic obstructive pulmonary disease;* *WBC,* *white blood cell count; RDW,* *red cell distribution width; BUN,* *blood urea nitrogen; ALT, alanine aminotransferase; AST,* *aspartate aminotransferase; PT,* *prothrombin time; APTT, activated partial thromboplastin time; TG, triglycerides; FBG, fasting blood glucose; TyG index, triglyceride glucose index.*
