# Supplementary material for: Triglyceride-glucose index as a potential predictor for in-hospital mortality in critically ill patients with intracerebral hemorrhage: a multicenter, case–control study
Source: BMC Geriatr. 2024 May 1;24:385. doi: 10.1186/s12877-024-05002-4 (PMC11061935; doi:10.1186/s12877-024-05002-4)
Supplement: Supplementary file 5 — Additional file 5. [file 12877_2024_5002_MOESM5_ESM.docx]

**Table.S3 The association between the TyG index and all cause in-hospital and ICU mortality in patients with ICH from eICU-CRD**

| **Variables** | **Model 1** | | |  | **Model 2** | | |  | **Model 3** | | |
| --- | --- | --- | --- | --- | --- | --- | --- | --- | --- | --- | --- |
|  | **OR（95% CI)** | ***P value*** | ***P-trend*** |  | **OR（95% CI)** | ***P value*** | ***P-trend*** |  | **OR（95% CI)** | ***P value*** | ***P-trend*** |
| **Hospital mortality** |  |  |  |  |  |  |  |  |  |  |  |
| Continuous variable  per unit | 1.67 (1.33,2.09) | <0.001 |  |  | 1.74 (1.38,2.19) | <0.001 |  |  | 1.37 (1.05,1.80) | 0.021 |  |
| Quantile **^a^** |  |  | <0.001 |  |  |  | <0.001 |  |  |  | 0.016 |
| Q1 (n=278) | Ref |  |  |  |  |  |  |  |  |  |  |
| Q2 (n=279) | 1.04 (0.60,1.80) | 0.900 |  |  | 1.02 (0.59,1.77) | 0.951 |  |  | 0.93 (0.52,1.70) | 0.826 |  |
| Q3 (n=278) | 2.10 (1.30,3.48) | 0.003 |  |  | 2.06 (1.27,3.41) | 0.004 |  |  | 1.54 (0.90,2.70) | 0.122 |  |
| Q4 (n=278) | 2.67 (1.67,4.37) | <0.001 |  |  | 2.82 (1.76,4.65) | <0.001 |  |  | 1.73 (1.02,3.06) | 0.036 |  |
|  |  |  |  |  |  |  |  |  |  |  |  |
| **ICU mortality** |  |  |  |  |  |  |  |  |  |  |  |
| Continuous variable  per unit | 2.01 (1.51,2.69) | <0.001 |  |  | 2.00 (1.49,2.68) | <0.001 |  |  | 1.61 (1.13,2.27) | 0.007 |  |
| Quantile **^a^** |  |  | <0.001 |  |  |  | <0.001 |  |  |  | 0.009 |
| Q1 (n=278) | Ref |  |  |  |  |  |  |  |  |  |  |
| Q2 (n=279) | 1.00 (0.42,2.36) | 0.993 |  |  | 1.00 (0.42,2.36) | 0.992 |  |  | 0.96 (0.39,2.37) | 0.924 |  |
| Q3 (n=278) | 2.40 (1.18,5.17) | 0.019 |  |  | 2.38 (1.17,5.13) | 0.020 |  |  | 1.61 (0.75,3.67) | 0.234 |  |
| Q4 (n=278) | 4.08 (2.12,8.52) | <0.001 |  |  | 4.00 (2.07,8.38) | <0.001 |  |  | 2.30 (1.09,5.16) | 0.034 |  |

***^a^*** *TyG index: Q1 (7.11–8.33), Q2 (8.33–8.76), Q3 (8.76–9.21), Q4 (9.21–12.44).*

*Model 1: unadjusted*

*Model 2: adjusted for age and gender*

*Model 3: adjusted for* *age, gender, GCS, hypertension, congestive heart failure, WBC, serum creatinine,* *serum BUN, serum bilirubin, serum AST, PT, anticoagulant agents, and antiplatelet agents*
