# Supplementary material for: Triglyceride-glucose index as a potential predictor for in-hospital mortality in critically ill patients with intracerebral hemorrhage: a multicenter, case–control study
Source: BMC Geriatr. 2024 May 1;24:385. doi: 10.1186/s12877-024-05002-4 (PMC11061935; doi:10.1186/s12877-024-05002-4)
Supplement: Supplementary file 7 — Additional file 7. [file 12877_2024_5002_MOESM7_ESM.docx]

**Table.S****4 Sensitivity analysis for the association between the TyG index and all cause ICU mortality ^a, b^**

| **Subgroup** | **MIMIC-IV** | | |  | **eICU-CRD** | | |
| --- | --- | --- | --- | --- | --- | --- | --- |
|  | **HR（95% CI)** | ***P value*** | ***P for interaction*** |  | **HR（95% CI)** | ***P value*** | ***P for interaction*** |
| All patients | 1.80 (1.27,2.56) | 0.001 |  |  | 1.46 (1.08,1.99) | 0.015 |  |
| Age |  |  | <0.001 |  |  |  | <0.001 |
| >60years | 3.10 (1.37,7.02) | 0.007 |  |  | 2.51 (1.58,4.32) | 0.005 |  |
| ≤60years | 1.65 (0.83,2.52) | 0.198 |  |  | 1.20 (0.69,2.11) | 0.518 |  |
| Diabetes |  |  | 0.028 |  |  |  | 0.427 |
| Yes | 1.74 (0.57, 5.30) | 0.328 |  |  | 1.30 (0.63, 2.69) | 0.476 |  |
| No | 1.92 (1.28, 2.86) | 0.001 |  |  | 1.52 (1.06, 2.19) | 0.023 |  |
| Hypertension |  |  | 0.008 |  |  |  | 0.045 |
| Yes | 2.17 (1.36,3.46) | 0.001 |  |  | 1.73 (1.16,2.63) | 0.006 |  |
| No | 1.76 (0.92,3.36) | 0.085 |  |  | 1.26 (0.77,2.19 ) | 0.067 |  |

**^a^** *Sensitivity analysis was conducted by the Cox proportional regression model.*

**^b^** *Cox regression model was adjusted for* *age, gender, GCS, hypertension, congestive heart failure, WBC, serum creatinine,* *serum BUN, serum bilirubin, serum AST, PT, anticoagulant agents, and antiplatelet agents.*
